# Culture Blind Leadership Research: How Semantically Determined Survey Data May Fail to Detect Cultural Differences

**DOI:** 10.3389/fpsyg.2020.00176

**Published:** 2020-02-18

**Authors:** Jan Ketil Arnulf, Kai R. Larsen

**Affiliations:** ^1^Department of Leadership and Organizational Behavior, BI Norwegian Business School, Oslo, Norway; ^2^Organizational Leadership and Information Analytics, Leeds School of Business, University of Colorado Boulder, Boulder, CO, United States

**Keywords:** latent semantic analysis, Likert scales, cross-cultural studies, organizational behavior, semantic versus empirical problems

## Abstract

Likert scale surveys are frequently used in cross-cultural studies on leadership. Recent publications using digital text algorithms raise doubt about the source of variation in statistics from such studies to the extent that they are semantically driven. The Semantic Theory of Survey Response (STSR) predicts that in the case of semantically determined answers, the response patterns may also be predictable across languages. The Multifactor Leadership Questionnaire (MLQ) was applied to 11 different ethnic samples in English, Norwegian, German, Urdu and Chinese. Semantic algorithms predicted responses significantly across all conditions, although to varying degree. Comparisons of Norwegian, German, Urdu and Chinese samples in native versus English language versions suggest that observed differences are not culturally dependent but caused by different translations and understanding. The maximum variance attributable to culture was a 5% unique overlap of variation in the two Chinese samples. These findings question the capability of traditional surveys to detect cultural differences. It also indicates that cross-cultural leadership research may risk lack of practical relevance.

## Introduction

A simple search for “cross-cultural leadership” through ISI Web of Science returns around 500 hits at the time this is written. An important source of empirical information in these appear to be survey methodology, mostly variations on Likert scale measures. At the same time, a recent methodological development has evolved that sheds a different light on the nature of such data. Relying on digital language algorithms, research on the Semantic Theory of Survey Response (STSR) has opened a way to predict survey patterns *a priori* based on the semantics of the survey items (Arnulf et al., 2014a, 2018a,b; Arnulf and Larsen, 2015; Nimon et al., 2015; Gefen and Larsen, 2017). An unintended but striking finding in one of these studies was that the semantic patterns computed in English were highly predictive also of survey patterns in a Norwegian sample, which raises an important question: If the statistical patterns in survey data are predictable across languages and cultures *a priori*, will such semantically driven surveys detect or neglect cultural differences?

The main tenet of STSR is that responses to survey items will correlate if the items share overlapping meanings. While this has been known and even intended to ensure consistency within scales, it would lead to contamination and inflated statistics if it happens between scales. Yet this is exactly what previous studies in STSR has found: Using algorithms for text analysis, up to 86% of the variance in relationships between commonly studied variables in leadership research were found to be predictable *a priori* (Arnulf et al., 2014a, 2018b; Nimon et al., 2015).

A peculiar implication of these findings is that if survey response patterns are caused by shared understanding of language, the same patterns should be detectable across languages to the extent that the items are correctly translated. Conversely, if the same survey do not create similar data patterns in samples from different cultures, the differences may be hard to explain even if it would be tempting to assume that differences in data structures are somehow caused by “culture.”

This study explores the extent to which cross-national response patterns to a leadership survey are predictable *a priori* through digital semantic algorithms. To achieve this purpose, we have used an instrument that has previously been found to demonstrate semantic predictability, and has also been widely used internationally, the Multifactor Leadership Questionnaire (MLQ) (Avolio et al., 1995; Bass, 1997, 1998). The study will cover native speakers of languages from English through Norwegian, German, Urdu and Chinese, and also compare the responses in native languages to responses in English from parallel respondent groups.

The study serves two purposes: Primarily, it seeks to establish the extent of variation in a cross-cultural leadership survey that can be attributed to semantic relationships. The inverse of this is the maximum amount of variation attributable to cultural factors in a wide sense of the term. Secondarily, this study raises a meta-theoretical question about how cross-cultural differences in leadership can be appropriately captured by our measurement instruments. Understanding the effect of language on leadership across cultures is of great importance in research as well as in practice (Hofstede et al., 2010; Gesteland, 2012; Mendenhall, 2013).

## Theory

The Semantic Theory of Survey Response (STSR) represents a new and hitherto unexplored aspect of survey data (Arnulf et al., 2014a, 2018b; Nimon et al., 2015). Briefly stated, STSR is not about the score levels of items – their purported measurements of latent variables. Instead, the focus of STSR is the semantic structure between the items of measurement instruments. If items in a study – or clusters of items in the form of subscales are semantically related, their mutual score pattern may be influenced by this. Purely semantic patterns in responses have been suggested earlier on theoretical (Feldman and Lynch, 1988; Schwarz, 1999) and experimental grounds (Michell, 1994). With the development of automated algorithms for text analysis, it is now possible to assess the impact and prevalence of this phenomenon in various domains of research (Larsen and Bong, 2016; Gefen and Larsen, 2017; Gefen et al., 2017).

Previous findings in STSR raise a number of methodological and theoretical concerns. What exactly does it imply if the correlation matrix of a survey instrument is predictable *a priori*? It is important here to note that we do not claim that score levels are predictable *per se*. What is predicted are the mutual relationships between the items. Due to the prevalent practice of structural equation modeling in fields like organizational behavior (OB), this means that the input data in the form of correlations or covariance matrix may according to STSR reflect semantic values instead of the purported attitude strength (for an in-depth treatment of this issues, see: Arnulf et al., 2018b, c).

The previous findings in STSR suggested that the factor structures of several instruments were predictable *a priori* due to heavy semantic influences. This is an empirical demonstration of a phenomenon argued conceptually in leadership research. van Knippenberg and Sitkin (2013) argued that the construct of transformational leadership is a tautology, where the dependent variable (leadership effectiveness) is already embedded in the definition and operationalizations of the dependent variables (leadership behaviors). The first study on STSR (Arnulf et al., 2014a) demonstrated empirically that this was in fact the case, and that the problem applied to other measures in leadership and motivation as well.

The meaning of semantic relationships in measurement terms can be understood through the way it works on scale coherence, usually expressed as Cronbach’s alpha. Items that share similar meanings (semantic overlap) tend to cluster around similar score levels. In a sense, they are not free to vary because their levels are dependent on each other – a person who believes that today is Friday is not semantically “free” to believe that tomorrow will be a Thursday. The previous studies on STSR found that despite the apparent independence of rotated factors, semantic relationships may still pervade (Arnulf et al., 2014a, 2018b). Measured constructs of leadership and motivation were found to relate semantically, albeit weaker than items within the scales. When this happens, the measured relationships between the latent variables are not free to vary but are mutually “locked.” Semantic relationships are not a universal characteristic of all such measurement instruments, as it was not strongly present in a personality inventory. That would imply that respondents to this measurement instrument are less restricted by their previous response in choosing the next response option (Feldman and Lynch, 1988; Maul, 2017; Arnulf et al., 2018c).

The nature and impact of semantic relationships are still not sufficiently understood. So far, we know that survey structures vary between almost complete semantic predictability to almost nothing at all (as in the case of the NEO personality inventory) (Arnulf et al., 2014a). It is likely that the phenomenon is more prevalent where the measures are reflective and the latent variables are social constructions (Arnulf et al., 2018d) than if the measures are formative (Arnulf, 2020). Several studies are going on to determine the variance components most influential in shaping semantic patterns, among others by applying multi-trait-multi-method (MTMM) approaches (Martinsen et al., 2017) but the picture is not yet conclusive.

What seems warranted to claim, however, is that to the extent that statistical patterns are predictable *a priori*, their empirical value is dubious since collecting them does not advance our knowledge (Smedslund, 1988, 2015; Semin, 1989; Elster, 2018). Semantically determined data patterns reflect agreements across interpretations of items that are common to most speakers. These will be the same across languages if the items in question are translatable.

The focus of STSR, then, is not on the actual score values themselves and the measures that they represent. Instead, STSR is concerned directly with the relationships among the variables – on item level and aggregated between scales.

This is a slightly different perspective from the traditional view on scores as inputs to, e.g., leadership surveys. Here, the score levels are usually collected for at least three purposes: Construct validation, empirical testing of theoretical hypothesized relationships between constructs, and for practitioners, to assess the presence of the theoretical phenomena in a given setting (Nunally and Bernstein, 2007; AERA et al., 2014; Slaney, 2017). For all three purposes, the responses are assumed to be expressions of attitude strength, as originally assumed by Likert (1932). In contrast, STSR is simply concerned with the predictability of semantic overlap between items, as earlier research has demonstrated how information about attitude strength is filtered out when the data structure is semantically determined (Arnulf et al., 2018b).

Culture usually serves as an important context that could presumably modify or even invalidate theoretical claims about leadership (House et al., 2004; Tsui et al., 2007; Mendenhall, 2013; Osland, 2013; Ma and Tsui, 2015). For that reason, the cultural validity of leadership constructs and their relationships to other OB constructs have received extensive attention during recent decades. There have also been a number of discussions about the methodological opportunities and pitfalls imminent in such research (House et al., 2004; Kirkman et al., 2006; Mansour et al., 2006; Hofstede et al., 2010). The present study does not aim at a comprehensive review of previously discussed opportunities and pitfalls. The focus here is on a specific problem with possibly wider ramifications: That cross-cultural research on OB may be trapped in semantic tautologies that obstruct real empirical insights.

### Semantically Determined Relationships

The Semantic Theory of Survey Response posits that the most obvious reason for correlations between survey items will be that they overlap in meaning (Arnulf et al., 2015). If a person thinks that today is Thursday, the person is also likely to think that tomorrow is Friday. This is not an empirical, but a semantic relationship – the one follows from the other (Semin, 1989; Smedslund, 1994; McEachrane, 2009). Ideas about weekdays may be blatantly obvious, but fuzzier examples of weaker relationships exist. People who say that they enjoy their jobs will also be less likely to look for new jobs – to want to keep a job is part of the meaning of liking one’s job. Since some people still look for other opportunities even while liking their present jobs, there will not be a perfect correlation between the two. These are examples of semantic relationships with various strengths.

“Semantics” is the branch of linguistics and logic concerned with meaning (Semin, 1989; Deerwester et al., 1990). The term “semantic relationship” usually implies one of two related meanings: Either the lexical definition of words and terms, as when using a dictionary, or the logical implication of one term from another as when explaining an argument. Until recently, semantics has been a domain for linguists and logicians. With the development of digital techniques for natural language processing, semantics has also become an important part of information technology (Landauer et al., 1998; Landauer, 2007; Dennis et al., 2013; Zhang et al., 2013). There now exist a variety of algorithms that can be used to index and compare the meaning of texts. Most readers are familiar with them in applications such as internet search engines. They can also be used for a number of advanced purposes such as automated translations or to establish ontologies – automated taxonomies that classify and organize knowledge about domains of discourse. Digital text algorithms can be used as tools to analyze and compare texts (Larsen and Bong, 2016; Gefen et al., 2017). They are relatively impartial in the sense that they follow transparent rules that will yield the same results across texts if applied in identical ways.

Using digital algorithms for text analysis, previous studies have found that widely used constructs within the OB domain are in fact semantically determined (Arnulf et al., 2014a, 2018a; Arnulf and Larsen, 2015; Nimon et al., 2015; Kjell et al., 2019). Digital algorithms take texts as their input and can perform computations on their meanings, comparing and grouping text according to quantitative measures of similarity. Digital algorithms have demonstrated the semantic link between constructs such as transformational leadership, LMX, 2-factor leadership, intrinsic motivation, OCB, and commitment (Arnulf et al., 2014a, 2018a). The specific semantic algorithms used in this study are further explained in the methods section.

The problematic side of semantic relationships is that they are basically only parallel or re-iterated versions of the same underlying propositions. This is easiest to see in the example concerning weekdays. If we know someone’s belief about which day we have today, we can predict all other statements that place the other 6 weekdays. It is also worth noticing that this is not limited to one language. The same sentences will be true in any other language as long as the language has words making up a 7-day week. That is because the propositional structure of the sentence is on a more abstract level than the words themselves. As long as the propositional structure is kept intact, the actual wording does not matter, whether within nor between languages.

While the example about the weekdays may be easy to understand, it gets harder when propositions only share some, but not all of their meaning. This is, however, the most likely reason for even weak correlations between survey items. If a respondent describes satisfaction with her job, the actual meaning of this is, among other criteria, that this job is preferable to other jobs. Hence, there is every reason to assume that job satisfaction will be negatively correlated with the intention to switch jobs. The correlation may however be far from perfect because “preferring this job to other jobs” is only one of many explanations for job satisfaction.

To the extent that survey data represent semantic relationships instead of attitude strength, they will not easily detect cultural differences. Most semantic relationships are translatable across modern languages and certainly in the field of organizations and leadership. To the extent that semantically determined correlations and other data structures are replicable across cultures and languages, it may only tell us that the semantic structure of the survey was correctly reproduced across these languages.

Therefore, Hypothesis 1:
Correlations in leadership surveys that are semantically determined in one language will be semantically predictable to a significant degree across all national languages and working environments.

### Cultural Differences in Survey Data

Conversely, if structures in survey data can be supposed to convey culturally determined patterns, they need to display variation that is unique to the linguistic or ethnic group as different from other, culturally unrelated samples (House et al., 2004). A simple version of this argument is frequently implied in the analysis of cross-cultural samples, in that differences between populations with different ethnic or other demographic characteristics are taken as indications of cultural similarities or differences.

A previous study has shown that while a range of respondent properties may influence score levels on leadership surveys, the ensuing correlation matrix has a tendency to converge around a structure predicted by semantics (Arnulf et al., 2018b). Our focus here is solely on the degree to which nationalities and languages influence the degree to which semantics can explain the item correlation matrix.

Languages pose a complex methodological challenge in research on management and OB (Harzing et al., 2011; Zander et al., 2011). The initial concern was to preserve the meaning of items when surveys were translated. Hence, it was suggested that surveys should be translated and independently translated back to assure that the meaning of the original items were preserved (Brislin, 1970; Herdman et al., 1997). More advanced developments in this field have recognized the insufficiency of this approach (Behr et al., 2016). While translation-back-translation may even create problems instead of solving them, a bigger problem arises when there is no accurate expressions in the second language for the target item of the original survey. For example, key modern-day English terms from the workplace do not necessarily exist or have the same meaning in other languages. The word “leadership” does not exist in, e.g., French, Italian or Japanese, but are usually substituted with the English word. The German counterpart for leadership (“Führung”) was politically contaminated and has largely been replaced with the English word “Management” (Arnulf et al., 2018d), but with slightly different meanings – what the linguists call “false friends” (Enfield, 2007).

While most survey items do not use such high-level concepts, they may still require the import of new linguistic constructions or professional expressions with limited public accept into the second language. In such cases, the survey may actually be translatable on one level and still difficult to understand at other levels (Behr et al., 2016). Differences in response statistics due to problems in understanding and translatability may appear as “cultural differences” but simply signal lack of understanding by the respondents.

Thus, Hypothesis 2:
Differences in survey response statistics between different ethnic and linguistic groups can be empirically explained by lack of understanding of the item texts, rather than systematic cultural differences.

#### Idiomatic Equivalence

While items may be accurately translated on a surface level, proper translations need to address the underlying propositional structure (Hanks, 1996; Behr et al., 2016). For example, a proverbial expression such as “to judge a book by its cover” is not actually about books, and is at the surface level easy to translate into any language that includes the concepts of ‘judgement’ and ‘books.’ If the underlying metaphorical phrase does not exist in the focal language or is less frequently used, respondents are less likely to fill out a survey appropriately. For example, translating the idiom to a language like Norwegian, will yield “å dømme en bok etter omslaget.” Many Norwegians will actually know of the English idiom, but a search for the phrase at Google.no will yield articles literally about whether consumers buy books based on the attractiveness of the cover. The requirement of idiomatic equivalence is common knowledge to most translators but it bears special relevance to the problem of semantic determination of survey response statistics (Arnulf et al., 2018d). If the translation departs from the idiographic essence, it can be inaccurate even when the superficial words look similar. In such cases, different statistics will not signal cultural differences but inaccurate translation.

The problem of idiomatic equivalence is therefore a core issue in cross-cultural leadership. Are different ways of conceptualizing work place phenomena simply different expressions of the same underlying theoretical “constructs,” or do they actually imply different cultural constructions of the work place? Only the latter case would indicate a true cultural difference, but it will be harder to detect within the conditions of the survey items itself. In this sense, survey data are “thin” in the sense of Geertz (1973) – they do not carry information about whether they are methodological artifacts or indicative of true cultural differences.

#### The Language Relativity Hypothesis

The proposition that native languages construct the experience in unique ways has had a long history in the humanistic and social sciences (Gumperz and Levinson, 1996). Most frequently attributed to Whorf (1956), there have been recurrent controversies about this topic (Lucy, 1996). The most extreme version of this hypothesis asserts that we do not experience what we have no words for, and conversely have richer experiences where we have more nuanced words. While this extreme version is probably not true (and also not endorsed by many), an increasing volume of empirical research seems to document that native languages do influence our cognitive functions and verbal interactions (Slobin, 1996; Boroditsky, 2011; Sidnell and Enfield, 2012; Gentner, 2016). A modified version of the linguistic relativity hypothesis seems to be documented and allow at least two important predictions: The first is that different languages provide different tools for perception and experiences. Language structures do not in themselves open or block experience, but they do guide attention and emphasis in culturally determined ways (Slobin, 1996). Languages are culturally accumulated tools and may be one of the most important sources of acculturation (Lakoff, 1987; Cavalli-Sforza, 2001; Pinker, 2008). While foreign language constructions may be expressible to some degree in every other language, the attention, nuances and importance of verbal content may be determined by one’s native language. Secondly, cognition and behavior in bilingual humans is influenced by the language in which they use in interactions (Hanks, 1996; Arnulf et al., 2014b). It follows from this that the most truly “culturally” determined responses detected in survey statistics are likely to be elicited from respondents to surveys in their native languages (Boroditsky, 2001, 2011; Boroditsky and Gaby, 2010; Fausey et al., 2010; Costa et al., 2017). Survey designs that use common corporate languages (usually English) may omit the translation problem, but will risk missing the truly “cultural” identity of a bilingual respondent. One way to ensure that differences in survey responses are truly culturally determined would be to combine two approaches, a native language and a corporate language approach. If the two conditions yield response patterns that are unique to the ethnic group, one may safely assume that it taps native language understanding while at the same time adheres to the same item structure that is presented to all participants (original language).

From the point of view of STSR, this sets up two criteria for determining cultural uniqueness in response patterns. First, the response pattern of the target group (e.g., Chinese) needs to be significantly less predictable by the language used in the algorithms (e.g., English). Second, there needs to be an identifiable shared proportion of variance between the target group surveyed in its native language (e.g., Chinese) and in the language used by the algorithm (e.g., English).

Thus Hypothesis 3:
Samples of respondents who do not have English as their native language will display unique common variance that is neither explained by semantic algorithms nor by response patterns from unrelated cultures.

In what follows, we will test the three hypotheses by applying text algorithms to a frequently used measurement instrument in leadership research and compare its predictive capabilities across a panel of diverse languages and ethnic groups.

## Materials and Methods

### Measures

#### Survey Instrument

The survey used for this study was the Multidimensional Leadership Questionnaire (MLQ) commonly used in research on transformational leadership (Tejeda et al., 2001; Piccolo et al., 2012). This instrument was used for two main reasons: For one, it has previously been shown to be semantically determined to a substantial degree (Arnulf et al., 2014a, 2018c). Secondly, it exists in a series of authorized non-English versions, frequently used in cross-cultural research and as basis for claims about cross-cultural validity of its main constructs^1^.

The MLQ was administered as a web-based survey, all items on a 5-point Likert scale and every item was fully labeled.

#### Semantic Algorithms

Following previous studies in STSR, we used two main types of algorithms. One is a corpus-based approach often termed MI (Mihalcea et al., 2006), the other is a vector-based approach called Latent Semantic Analysis (LSA) (Deerwester et al., 1990). These algorithms are extensively published and described methodologically elsewhere in articles on semantics in psychometrics (Arnulf et al., 2014a, 2018a; Larsen and Bong, 2016; Gefen and Larsen, 2017; Gefen et al., 2017), but their main features are presented briefly here.

The MI algorithm (Mihalcea et al., 2006) extracts meaning from a lexical database called WordNet (Poli et al., 2010). It parses sentences into words and detects part-of-speech to better detect the correct category for the words in WordNet. Word specificity refers to the specific meaning of words (e.g., collie and sheepdog) versus generic concept words (e.g., animal and mammal). Specific words are given higher weight than abstract concepts (such as animal). The British National Corpus (Sparck-Jones, 1972) is used to calculate inverse document frequency (Sparck Jones, 1986). The version of the MI algorithm used here is the same as that used in Larsen and Bong (2016), which along with path similarity averages word-similarity metrics from Wu and Palmer (1994), Jiang and Conrath (1997), and Lin (1998). These metrics were created to measure word relatedness and similarity by calculating the shortest distance between given words’ synsets (sets of synonymous words) in the WordNet hierarchy; the shorter the distance between words, the higher the similarity score. For implementation details on the MI algorithm, please see Larsen and Bong (2016).

Through a combined calculation of lexical distances and the syntactic structure of the sentences, the MI algorithm will assign a number signifying overlap in meaning between any two survey items (Mihalcea et al., 2006). This number will always be between 0 and 1.00, where a higher number indicates greater overlap of meaning. The numbers are structurally similar to correlations but cannot take negative values and are also different from correlations in that they do not depend on co-variation– they are strict assessment of the overlap of meaning.

The LSA algorithm does not make any use of pre-defined lexical information. Instead, it “extracts” meaning from large samples of existing text called “semantic spaces” (Dennis et al., 2013; Gefen et al., 2017). These semantic spaces are made up of hundreds of millions of words that have been collected from a defined text universe, such as newspaper articles, textbooks or scientific publications. These text samples are turned into a word-by-document matrix, then further reduced in a statistical technique called “singular value decomposition” (SVD). The similarity of texts such as survey items can then be determined by projecting the items texts onto the SVD-transformed matrices (Gefen et al., 2017). The output from LSA are the cosines of the compared items in these matrices. Like the MI values, the LSA values usually fall in the range between 0 and 1.00 even though they occasionally do take negative values. These negative values are however not the same as negations.

All these algorithms are still inferior to humans in their ability to detect meaning (Landauer, 2007). Since the LSA output is dependent on the semantic space applied, we usually compute LSA values from multiple semantic spaces to approximate the understanding of human speakers. Finally, by combining MI and LSA values in multiple regression, we can approximate the semantic understanding of human subjects as a combination of lexical and domain-specific knowledge, as shown by previous authors (Arnulf et al., 2014a, 2018a). As will be discussed below, the semantic algorithms are still inferior to language parsing in humans. While the data sources (WordNet and newspaper articles) used in the algorithms are not unbiased (see, for example Baeza-Yates, 2018), none of these sources were designed or collected with knowledge that they would one day be used to evaluate survey items.

Despite their shortcomings, the algorithms pose a sort of “impartial” standard for semantic structures in that they are transparent and completely rule-based, leaving out subjective measurement errors (Stark, 2018).

#### Human Respondent Samples

Because of the cross-cultural, multi-language nature of this study, we aimed to obtain a broad and still balanced set of sub-samples. The semantic algorithms were all computed in English and the prevalently used leadership survey MLQ was also originally published in English. Hence, we chose English as the basic language of the analysis. This is also in line with a prevalent practice of using English as corporate language across the world (Harzing et al., 2011; Zander et al., 2011).

We sought to compare groups with native languages of differing distance to English, ranging from proximal to distant in terms of language families. We obtained one sample of 146 native speakers of English to represent the baseline computed by the algorithms. The samples with native languages closest to English were obtained in Norwegians (*N* = 1,226 sampled in Norwegian and 180 Norwegians responding in English) and Germans in German (*N* = 59, none in English). These languages share the Indo-European language roots of English and are assumed to be distinct but close (Cavalli-Sforza, 2001). As a more remotely related sample, we chose Pakistanis responding in Urdu (*N* = 111) and Pakistanis responding in English (*N* = 108). Urdu is another Indo-European language but with much more distant relationship to English than the other two (Cavalli-Sforza, 2001). Finally, we chose Chinese (*N* = 259 Chinese responding in Mandarin and 240 Chinese responding in English) as the sample with the greatest linguistic and cultural distance from English (Needham and Harbsmeier, 1998; Cavalli-Sforza, 2001; Norenzayan et al., 2002). Through the data sampling procedure (see below) we also had three other mixed sub-samples: 45 other Europeans responding in English, 49 Indian nationals in English (who stated other options as their native language, e.g., Tamil, Malayalam, etc.), and 58 non-Chinese East Asian citizens responding in English (mostly Indonesians, Koreans, and Japanese).

The data mainly stem from leadership surveys carried out in four globally present companies. The employees from these companies were mainly staff working with banking, engineering, sales and administrative functions such as accounting and HR. The responses were mostly sampled from locations in Norway, Dubai, India, Singapore, Korea and China. To balance the design, there were three convenience samples: The native speakers of German and about a third of the native speakers of English were recruited through the network of the researchers. The native English speakers were a mixed group of people from the United States and the United Kingdom, with a small number of Indian and Singaporean citizens who described their native languages as “English.” Half of the Pakistani respondents using Urdu were working at an engineering college in Pakistan, but another half were first generation immigrants in Norway working in diverse professions.

For the whole sample, the mode of the age group was 35–44 years, and 58% were male. While 68.1% described themselves as non-managers, 25.1% were middle managers, 4.1 were upper management and 2.7% described themselves as executive level.

### Analytical Strategy

As previously stated, our analysis aims at exploring the degree to which the observed item response matrices (the dependent variables) of our various samples are explained in regression equations using the semantic indices as independent variables.

## Results

We first established the characteristics of each sample in terms of demographics, linguistic background and the main score levels on the leadership scales of the MLQ. Table 1 presents these values in overview.

**TABLE 1:** Sample characteristics and score levels.

			**Mean leadership score levels**			
			
**Experimental group**	***N***	**Male/Female**	**Transformational leadership**	**Transactional leadership**	**Laissez-faire**	**Outcome scores**
English native speakers	146	70%/30%	3.4	2.9	2.0	3.5
Norwegians in Norwegian	1226	51%/49%	3.7	3.0	2.1	3.6
Norwegians in English	180	82%/18%	3.5	2.6	1.7	3.7
Germans in German	59	61%/39%	3.3	3.1	2.3	3.5
Other Europeans in English	45	80%/20%	3.6	2.9	1.9	3.6
Pakistanis in Urdu	111	n/a	3.7	3.5	3.2	3.8
Pakistanis in English	108	n/a	3.7	2.8	1.9	4.1
Indian nationals in English	49	82%/18%	3.4	2.9	1.9	3.5
Chinese in Chinese	235	57%/43%	3.5	3.0	2.0	3.5
Chinese in English	240	61%/39%	3.6	3.0	1.7	3.7
East Asians in English	58	76%/24%	3.6	3.0	1.9	3.6
**Total dataset**	**2513**	**58%/42%**	**3.6**	**3.0**	**2.1**	**3.6**

An ANOVA analysis shows that the differences in score levels between the samples are statistically significant, but not large. For all samples, the transformational leadership score averages are in the range of 3.3 – 3.7. The score levels of transformational leadership are universally higher than the sample scores for transactional leadership, where the range is wider (2.6 – 3.5). The range of Laissez-faire is 1.7 – 3.2, and the outcome scores range between 3.5 and 4.1. More importantly, the differences in means appear to be random variation without any systematic relation to sample size or cultural distance from native speakers of English.

The full version of the MLQ contains 45 items (Avolio et al., 1995). This turns into a matrix of (45^∗^44)/2 = 990 unique item correlations. The semantic method addresses these relationships, which are also important to most prevalent statistical models. The correlations or co-variances between items and scales are commonly used to build statistical models in survey research (Jöreskog, 1993; Borsboom, 2008; MacKenzie et al., 2011; Lamiell, 2013; Slaney, 2017; Van Dierendonck et al., 2017). To the extent that these are semantically determined, the semantic influence will be retained in all subsequent models.

We therefore regressed the semantic values on the item correlation matrix for each sample. This can be done in three ways (Arnulf et al., 2018a): The first is a multiple linear regression where we use all the semantic information but in a purely linear model. This approach probably underestimates the semantic influence, because the semantic algorithms available at present cannot take context into consideration. Human speakers use context as an important signal to differentiate between different meanings of the same words. To emulate this, we may set up a general linear model (GLM) that allows the equation to “know” which scale any item belongs to. This comes close to human contextual understanding and is justified because the scale belongingness is significantly predictable by the algorithms (Arnulf et al., 2014a). We try two types of GLM: In the first model, we only use the main effects on the variables but set the constants as fixed within the scales. In the second model we use the full interactions between the variables. The final approach obviously risks overfitting the model. We therefore report the results of all three models, taking the linear model as a lower and the GLM estimates as an upper limit to the “true” effect of semantics on the correlation matrix.

Hypothesis 1 stated that “Correlations in leadership surveys that are semantically determined in one language will be semantically predictable to a significant degree across all national languages and working environments.” This is tested and listed for each of the language subgroups in Table 2.

**TABLE 2:** Predicted variation of the correlation matrix for each linguistic sub-sample, compared with a principal component analysis (PCA) of each sample.

**Experimental group**	**Predicted in linear regression (adj *R*^2^)**	**Predicted in GLM (adj *R*^2^)**	**Predicted in GLM full factorial (adj *R*^2^)**	**PCA Eigenvalues > 1**	**Variance explained by the PCA factors**	**PCA Visual Scree factors**
English native speakers	0.84	0.87	0.91	7	70	1
Norwegians in Norwegian	0.79	0.86	0.91	6	59	1
Norwegians in English	0.66	0.77	0.89	11	71	1
Germans in German	0.67	0.73	0.80	9	75	3
Other Europeans in English	0.77	0.83	0.94	8	82	3
Pakistanis in Urdu	0.11	0.21	0.31	12	72	5
Pakistanis in English	0.43	0.55	0.71	11	76	3
Indian nationals in English	0.73	0.78	0.83	8	78	1
Chinese in Chinese	0.54	0.59	0.67	10	69	2
Chinese in English	0.72	0.77	0.86	10	67	3
East Asians in English	0.55	0.67	0.74	10	85	2
Total dataset	0.79	0.85	0.92	6	57	3

All regression models are significant (*p* < 0.001), and therefore support hypothesis 1. However, there are differences that could conceivably be due to culture. The same semantic values predict the different linguistic groups in a range from 84% in the case of native English speakers down to 11% for Pakistanis responding to a version in Urdu. In fact, there is a strong negative relationship between semantic predictability and the complexity of the factor structure when the samples are subjected to a principal component analysis (PCA): The more semantically predictable the dataset appears to be, the lower the number of Eigenvalues above 1 and the lower the number of factors visually identifiable in the Scree plots.

While this could indicate different cultural backgrounds in leadership cultures, the more parsimonious interpretation is that it could be noise due to lack of understanding. There are particularly three conspicuous facts that point in this direction: The Norwegians are strongly semantically predictable, but more predictable in their native language Norwegian than in English. The Pakistanis seem only vaguely compliant with the semantics when answering in Urdu, but much more so for those who are allowed to answer in English. The two Chinese samples, that linguistically and culturally should be more distant from the Anglo-Saxon culture than the Pakistanis, are much more influenced by semantics and also here, those surveyed in English seem more semantically predictable than those responding in Chinese. Moreover, the Indian nationals, who arguably are not culturally very distant from the Pakistanis, are very semantically predictable when responding in English. In short, there does not seem to be a systematic pattern that explains how samples depart from the semantically expected.

Using the *R*^2^’s tells only part of the story. If the departure from semantically expected correlations are due to noise, the residuals will be fairly random, and the systematic part of the variation will still be semantics. The first way to test this is to see how well the semantically predicted correlations actually match the real survey correlations. Central to leadership research is an interest in the mutual impact of leadership behaviors on purported outcomes (March and Sutton, 1997; Dumdum et al., 2002; Hansen et al., 2013; Arnulf et al., 2018d). Since the MLQ contains a separate scale for outcomes, we can average the correlations between each leadership behavior and the outcome measures and compare these to the values predicted in the respective regression models. We can thereby estimate how the semantic values predict the theoretically proposed relationships between leadership behaviors and outcomes in the employees. This is displayed in Table 3.

**TABLE 3:** Average correlations between leadership scales and the outcome measures, with their semantically predicted counterparts, by linguistic sub-sample.

**Experimental group**	**Conditional reward**	**Individ. con-sideration**	**Idealized influence attr.**	**Idealized influence beh.**	**Inspiring motivation**	**Intellect. stimulation**	**Laissez-faire**	**Active mgmnt by except.**	**Passive mgmnt by except.**	**Outcome to outcome**	**All other relationships**	**Avg residuals**
English native speakers	0.55	0.54	0.57	0.48	0.54	0.53	−0.45	0.26	−0.32	0.70	0.16	
*Predicted in linear regr.*	*0.48*	*0.52*	*0.48*	*0.45*	*0.48*	*0.46*	−*0.35*	*0.45*	−*0.34*	*0.56*	*0.17*	*0.07*
***Predicted in GLM***	*0.52*	*0.54*	*0.53*	*0.46*	*0.51*	*0.49*	−*0.39*	*0.33*	−*0.27*	*0.70*	*0.16*	*0.03*
Norwegians in Norwegian	0.47	0.54	0.52	0.51	0.52	0.50	−0.36	0.16	−0.19	0.60	0.18	
*Predicted in linear regr.*	*0.43*	*0.48*	*0.45*	*0.44*	*0.47*	*0.43*	−*0.25*	*0.42*	−*0.25*	*0.53*	*0.19*	*0.08*
***Predicted in GLM***	*0.47*	*0.52*	*0.48*	*0.49*	*0.50*	*0.47*	−*0.31*	*0.23*	−*0.16*	*0.60*	*0.18*	*0.03*
Norwegians in English	0.41	0.46	0.55	0.37	0.45	0.47	−0.37	−0.03	−0.26	0.63	0.13	
*Predicted in linear regr.*	*0.36*	*0.40*	*0.37*	*0.35*	*0.37*	*0.35*	−*0.23*	*0.34*	−*0.23*	*0.45*	*0.14*	*0.11*
***Predicted in GLM***	*0.37*	*0.44*	*0.44*	*0.35*	*0.39*	*0.40*	−*0.28*	*0.10*	−*0.16*	*0.63*	*0.13*	*0.06*
Germans in German	0.49	0.55	0.52	0.40	0.48	0.48	−0.41	0.15	−0.15	0.64	0.17	
*Predicted in linear regr.*	*0.39*	*0.45*	*0.42*	*0.41*	*0.45*	*0.40*	−*0.21*	*0.39*	−*0.20*	*0.49*	*0.18*	*0.10*
***Predicted in GLM***	*0.43*	*0.49*	*0.46*	*0.39*	*0.47*	*0.39*	−*0.28*	*0.24*	−*0.09*	*0.64*	*0.17*	*0.05*
Other Europeans in English	0.53	0.57	0.66	0.58	0.53	0.63	−0.57	0.08	−0.39	0.69	0.15	
*Predicted in linear regr.*	*0.50*	*0.54*	*0.50*	*0.46*	*0.47*	*0.48*	−*0.41*	*0.46*	−*0.41*	*0.59*	*0.16*	*0.11*
***Predicted in GLM***	*0.53*	*0.57*	*0.59*	*0.52*	*0.49*	*0.52*	−*0.49*	*0.22*	−*0.32*	*0.69*	*0.15*	*0.05*
Pakistanis in Urdu	0.18	0.20	0.28	0.18	0.22	0.25	0.19	0.12	0.08	0.08	0.38	
*Predicted in linear regr.*	*0.18*	*0.22*	*0.22*	*0.22*	*0.23*	*0.24*	*0.22*	*0.11*	*0.21*	*0.11*	*0.25*	*0.04*
***Predicted in GLM***	*0.20*	*0.26*	*0.17*	*0.21*	*0.23*	*0.16*	*0.11*	*0.16*	*0.08*	*0.38*	*0.18*	*0.09*
Pakistanis in English	0.14	0.35	0.34	0.44	0.30	0.46	0.30	−0.17	−0.12	−0.22	0.57	
*Predicted in linear regr.*	*0.15*	*0.26*	*0.32*	*0.30*	*0.30*	*0.32*	*0.28*	−*0.10*	*0.27*	−*0.10*	*0.36*	*0.11*
***Predicted in GLM***	*0.27*	*0.35*	*0.33*	*0.28*	*0.35*	*0.27*	−*0.09*	*0.04*	−*0.11*	*0.57*	*0.14*	*0.22*
Chinese in Chinese	0.37	0.33	0.34	0.38	0.42	0.45	−0.32	0.18	−0.10	0.53	0.18	
*Predicted in linear regr.*	*0.34*	*0.38*	*0.35*	*0.36*	*0.40*	*0.34*	−*0.13*	*0.35*	−*0.12*	*0.40*	*0.18*	*0.07*
***Predicted in GLM***	*0.35*	*0.32*	*0.32*	*0.38*	*0.39*	*0.38*	−*0.24*	*0.22*	−*0.08*	*0.53*	*0.18*	*0.03*
Chinese in English	0.42	0.33	0.41	0.39	0.41	0.43	−0.26	0.19	−0.22	0.56	0.16	
*Predicted in linear regr.*	*0.37*	*0.37*	*0.34*	*0.34*	*0.36*	*0.34*	−*0.16*	*0.34*	−*0.16*	*0.40*	*0.16*	*0.07*
***Predicted in GLM***	*0.39*	*0.31*	*0.37*	*0.36*	*0.37*	*0.38*	−*0.18*	*0.23*	−*0.13*	*0.56*	*0.16*	*0.04*
Indian natives in English	0.51	0.45	0.63	0.44	0.58	0.52	−0.47	0.25	−0.29	0.61	0.17	
*Predicted in linear regr.*	*0.49*	*0.51*	*0.47*	*0.44*	*0.45*	*0.45*	−*0.33*	*0.45*	−*0.33*	*0.54*	*0.17*	*0.08*
***Predicted in GLM***	*0.51*	*0.48*	*0.57*	*0.42*	*0.53*	*0.47*	−*0.42*	*0.32*	−*0.25*	*0.61*	*0.17*	*0.03*
East Asians non-Chinese English	0.46	0.42	0.46	0.52	0.53	0.46	−0.11	0.13	0.06	0.58	0.26	
*Predicted in linear regr.*	*0.39*	*0.44*	*0.42*	*0.43*	*0.46*	*0.40*	*0.00*	*0.40*	*0.01*	*0.47*	*0.26*	*0.08*
***Predicted in GLM***	*0.40*	*0.43*	*0.45*	*0.48*	*0.50*	*0.42*	−*0.09*	*0.22*	*0.10*	*0.58*	*0.26*	*0.03*
Whole dataset correlations *N* = 2513	0.18	0.44	0.45	0.48	0.44	0.49	0.47	−0.31	0.13	−0.18	0.59	
*Predicted in linear regr.*	*0.18*	*0.39*	*0.43*	*0.40*	*0.40*	*0.43*	*0.39*	−*0.20*	*0.38*	−*0.19*	*0.48*	*0.07*
***Predicted in GLM***	*0.18*	*0.42*	*0.44*	*0.43*	*0.42*	*0.45*	*0.42*	−*0.26*	*0.21*	−*0.14*	*0.59*	*0.03*

The overview shows that the correlations between the various leadership behaviors and the outcome values are almost equally well predicted across the linguistic sub-samples, ranging from almost identical in the case of GLM to somewhat less precise in linear regression. The finding is in accordance with the theoretical tautology problem pointed out by van Knippenberg and Sitkin (2013) as the relationships between independent and dependent variables are semantically determined. One important finding however is that the residuals – or precision – of the predicted correlations is almost independent of the adjusted *R*^2^ in each sample. The proportion of variance explained by semantics predicts only 3% of the variance in the residuals from linear regression from sample to sample. In other words, the non-semantic information is mostly noise, so that most of the signal is determined by the semantics – if there are relationships, these are most likely to be produced by semantics.

This is in line with hypothesis 2, which stated that “differences in survey response statistics between different ethnic and linguistic groups can be empirically explained by lack of understanding of the item texts.” While this is not in itself a clear test of Hypothesis 2, this will be subjected to further testing below. However, we first want to test Hypothesis 3. This stated that “Samples of respondents who do not have English as their native language will display unique common variance that is neither explained by semantic algorithms nor by response patterns from unrelated cultures.”

To identify the uniquely ethnic variance components in the data, we applied a stepwise hierarchical regression analysis, implying the following theoretical considerations: As argued initially, we assume that Chinese natives responding in Chinese will be most likely to display cultural differences from the native English speakers. We therefore enter the semantic similarity indices in the first block as the undisputedly semantic predictors of variance. As mentioned, the digital algorithms are still inferior to most adult human speakers in parsing semantic structures. In the second step, we therefore enter the values for native speakers of English. To the extent that these numbers express something in common with the native Chinese speakers, it should be something like the knowledge common to all humans with no special cultural significance. Further, we add Norwegians and Germans in their native languages in step 3, as there is no reason either to think that these groups share cultural characteristics with Chinese. In step 4, we add Norwegians and other Europeans in English. In step 5, we enter Pakistanis and Indian nationals in English, as we are now moving eastwards in cultural influence. In step 6, we enter non-Chinese East Asians in English. In step 7, we finally enter the Chinese responding in English. This allows us to inspect if the explained variance increases as we add samples with more Asian cultural elements. The result is displayed in Table 4.

**TABLE 4:** Predicting Chinese outcome patterns in hierarchical regression by semantics and other subgroups.

**Cultural influence**	**Models**					**Adjusted *R*^2^**	**Adjusted *R*^2^ increase**	**df**	**Mean square**	***F***
Algorithm block	(1) Semantic algorithms alone					0.54		4	12.03	287.57
European language block		(2) Adding native English speakers				0.63	0.09	5	11.28	337.18
		(3) Adding Norwegians and Germans in their native languages				0.64	0.01	7	8.17	250.11
		(4) Adding Norwegians and other Europeans in English				0.66	0.02	9	6.59	215.08
Indian subcontinent			(5) Adding Indian and Pakistani natives in English			0.69	0.03	11	5.60	197.79
East Asian				(6) Adding non-Chinese East Asians in English		0.73	0.04	12	5.44	221.70
Uniquely Chinese					(7) Adding Chinese in English	0.77	0.05	13	5.34	261.09

**P*-values for all models and increases in *R*^2^ < 0.001.*

Hypothesis 3 seems supported in that there is a unique component of variance comprising 5% that is shared only between the two Chinese samples responding in either Chinese or English.

However, the uniquely Chinese variance seems small. The bulk of variance seems predicted by the semantic algorithms alone (54%). Adding native English speakers and Europeans improve the prediction by 12%, reaching 66% with no probable influence from uniquely Chinese cultural heritage. There is an arguable Asian component in between – 3% from the Indian subcontinent or 4% from the non-Chinese East Asians.

The sample with the most deviant statistical pattern does however seem to be the Pakistanis responding in Urdu, not the Chinese as theoretically expected. We again tried the same stepwise regression to see if there is a uniquely Pakistani way of responding to the MLQ. As in the previous model, we entered the semantics and the native English speakers first. This time though, the Indian natives came toward the end, before the Pakistani sample in English was entered in the model.

As can be seen from Table 5, the uniquely Pakistani variance component (i.e., shared only between Pakistani respondents in Urdu and in English) is at most 3%. They do not share any unique variance at all with Indian natives.

**TABLE 5:** Predicting Pakistani outcome patterns in hierarchical regression.

**Cultural influence**	**Models**						**Adjusted *R*^2^**	**Adjusted *R*^2^ increase**	**df**	**Mean square**	***F***	
Algorithm block	(1) Semantic algorithms alone						0.11		4	0.66	31,92	
European language block		(2) Adding native English speakers					0.20	0.09	5	0.94	5.25	
		(3) Adding Norwegians and Germans in their native languages					0.20	0.00	7	0.68	36,60	ns
		(4) Adding Norwegians and other Europeans in English					0.25	0.05	9	0.64	35,85	
East Asian			(5) Adding Chinese in Chinese				0.25	0.00	10	0.57	32,25	ns
				(6) Adding Chinese and non-Chinese East Asians in English			0.26	0.01	12	0.49	27,85	
Indian subcontinent					(7) Adding Indian Natives in English		0.26	0.00	13	0.46	25,83	ns
Uniquely Pakistani						(8) Adding Pakistanis in English	0.29	0.03	14	0.48	28,12	

To intensify the analysis of the seemingly aberrant statistics from Pakistanis in Urdu, we did a further breakdown of the dataset. 65 of the Urdu responses were collected in Pakistan and another 46 responses were collected among first generation immigrants to Norway. We repeated a stepwise regression model, entering only semantics and Pakistanis in English first, but this time tried to analyze how much unique variation the two different Urdu samples seemed to have. The results are displayed in Table 6, and it turns out that the two different Urdu samples have absolutely nothing uniquely in common.

**TABLE 6:** The Urdu samples from Pakistan and Norway in hierarchical regression.

**Cultural influence**	**Models**				**Adjusted *R*^2^**	**Adjusted *R*^2^ increase**	**df**	**Mean square**	***F***	
Algorithm block	(1) Semantic algorithms alone				0.03		4	0.36	8,0.0	
European language block		(2) Adding native English speakers			0.05	0.02	5	0.50	11.86	
Pakistanis in English			(3) Adding Pakistanis in English		0.06	0.01	6	0.50	11.99	
Uniquely Urdu				(4) Adding Pakistanis from Norway in Urdu	0.06	0.00	7	0.44	10.41	ns

As argued in our initial discussion, we suspected that something was wrong with the Urdu translation of the MLQ, or with the samples, and we therefore contacted a certified Urdu translator who judged the materials. He could quickly give us a likely explanation for the chaotic statistics. Many Pakistani citizens will actually not have Urdu, but Punjabi as their native language. However, while Urdu is also a written language, Punjabi is only an oral language, a fact corroborated by a linguistic report on Pakistanis in Norway (Thiesen, 2003). Many Pakistanis will therefore claim that their native language is Urdu, even if this is strictly not correct. The most likely reason for the noisy statistical patterns is therefore simply a lack of understanding – the respondents have inadequate reading skills in Urdu. We take this as support for hypothesis 2, claiming that lack of linguistic proficiency is the most likely cause of reduced semantic predictability where this is elsewhere found to be substantial. A further corroboration of this interpretation can be found by comparing the Norwegians responding in Norwegian to the Norwegians responding in English. Since the English survey version among English native speakers is the most semantically predictable condition, the lack of semantic predictability of Norwegians is probably due to the difference in their proficiency in English and their native language. Lack of proficiency in English is the best explanation for the drop in semantic predictability.

**FIGURE 1:**
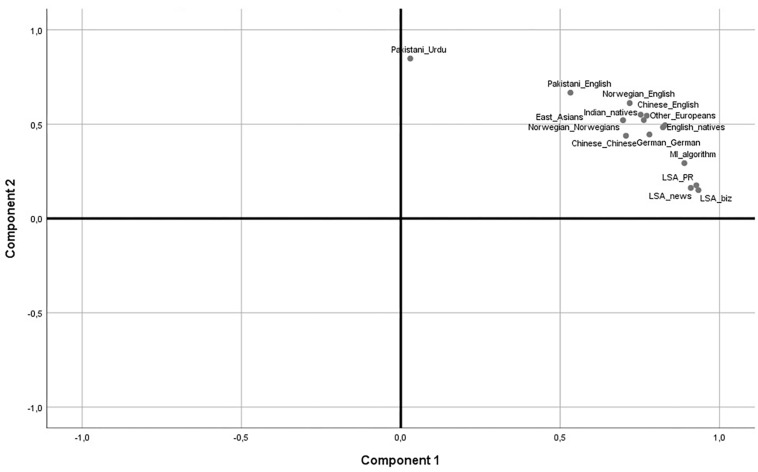
Linguistic sub-samples and semantics in rotated 2 factor PCA.

As a final check, we subjected all the 990 item pair correlations for each linguistic sub-sample with the semantic values to a PCA with varimax rotation. This is a procedure used earlier to separate and map languages and genes according to anthropological developments, and tends to yield clusters of related languages (Cavalli-Sforza, 2001). The PCA displayed two factors, displayed as a 2-factor plot in Figure 1. It can be seen that one factor is essentially made up of the sample responding in Urdu. The rest of the sample clusters unsystematically around the semantic values created by the algorithms. Thus, there are no signs that the responses in Urdu are culturally determined, but most likely a result of inadequate language skills. Also, the two-dimensional plot supports H1 in that the overwhelming determinant of variation in the data is semantic.

## Discussion

The purpose of this study was to explore the extent to which semantic algorithms can predict correlation matrices across different languages and national samples in a semantically determined leadership survey. It was theorized that the propositional structures inherent in semantic information are largely translatable across languages.

This study administered a globally prevalent leadership survey with established semantic properties to a broad cross-cultural sample spanning the Anglo-Saxon cultural domain (native English speakers), northern Europe (Norwegians and Germans), the Indian subcontinent (Pakistani and Indian natives) as well as East Asia (China, Korea, Indonesia, Malaysia and Japan).

For all sub-samples, the semantic algorithms predicted significant proportions of the variation in correlations between items, ranging from 11 to 84%. The semantic algorithms were computed using the English version of a survey originating from the United States. It is therefore natural that the best predicted sample was the native speakers of English (mostly United States and United Kingdom citizens).

The next best prediction occurred also mostly for samples responding in English. This was true for non-English speakers from Europe, Indian nationals and even Chinese respondents in English. The differences in statistical patterns are therefore largely attributable to linguistic precision and understanding. One interesting example is provided by the two Norwegian samples. Norwegian is a Germanic language close to English (Renfrew, 1987; Cavalli-Sforza, 2001), and Norwegians are usually quite competent speakers of English (Warner-Søderholm, 2013). There is no wonder therefore, that both samples seem fairly semantically determined. As the Norwegian sample responding in English is slightly less semantically predictable than the one responding in their native language may therefore be due to a lack of linguistic precision. The Norwegian language version of the MLQ may be quite adequate, and better than the private translation that a Norwegian respondent needs to do while responding to a version in English.

For samples with a more remote relationship to English, there may be other explanations. Chinese and Pakistani nationals respond much more semantically driven when responding to an English version of the scales than to versions in their own native languages.

To the extent that survey data are sensitive to cultural differences, such effects should arguably be most likely to occur in the non-English speaking samples responding in their native languages (Boroditsky, 2011; Harzing et al., 2011; Zander et al., 2011). Hence, it is natural that the semantic algorithms show lesser predictive values for these than to other samples. However, it is hard to say what these differences in response patterns may imply (cfr. Russell, 1922; Behr et al., 2016). A comparison of the predicted correlations with the observed ones showed that these were fairly close even in the case where semantics predicted only weakly. This is a finding akin to earlier findings in studies of variance components in responses and semantic predictability, in that the semantic patterns are the main driver of the observed correlations (Arnulf et al., 2018b). If other variance components exerted notable influence, the English language semantic values should be systematically off target the more culturally disparate the sub-sample was. This did not seem to be the case. Among the respondents in the sample, there were obviously groups very different from the native English speakers. Still, the response patterns were notably influenced by semantics as predicted by the algorithms.

The strongest deviations from the semantic patterns were found in the Pakistani sample responding in Urdu. The two Urdu samples, the one in Pakistan and the one in Norway, had no shared variation, and did not share unique variation with either other Pakistanis in English or the sub-sample from the Indian subcontinent that would be their most likely cultural relative. Everything considered, the statistics in the Urdu samples were most likely influenced by problems with the translation of the survey and even more by inadequate reading capabilities in the respondents. This is also in line with other research that has replicated the variable structure of transformational leadership in Pakistan (Khan et al., 2014).

This study made the theoretical claim that Chinese responding in Chinese should appear as culturally most distant to the native English speakers. If we disregard the obvious language problem in the Urdu group, the Chinese responding in Chinese did display the lowest semantic predictability in the study, as expected. However, when we controlled for all non-Chinese speakers, there was not much unique variation left among the Chinese respondents. The two Chinese samples responding in English and Chinese shared only 5% unique variation, less than a tenth of the variation they shared with the numbers from the digital algorithms. The unique variations between the ethnic samples in the native/English conditions were always around one to five percent, which may well be within random range. This shared variation was of the same magnitude as the differences within the non-Asian samples and within Asia. There are no compelling reasons to attribute these differences to cultural similarities between Chinese and Indians, or between Chinese and Japanese for that matter (Wang and Satow, 1994; Liu et al., 2004; Aoki, 2008).

A recent study on significant differences between score levels of groups has indicated that even with notable *p*-values and effect sizes, similarities in group distributions may practically outweigh the noted difference substantially (Hanel et al., 2019). The study proposes a measure called absolute effect (AE), defined as the median difference expressed as the percentage of the largest possible scale difference. Exploring the Semantic Theory of Survey Response we are usually not concerned with the score levels *per se*. Instead, we are investigating how the mutual patterns among survey responses reflect semantically given structures. If we apply the rationale behind the AE on the semantic structure in our study, a 5% shared unique variance among Chinese respondents equals an average “freedom” in responses in the MLQ of 5% of at most 0.2 scale points on a 5-scale Likert scale option. Or stated differently, the median Chinese respondent may be expected to depart 0.2 Likert scale score points from an English native speaker. The practical impact of this is hard to grasp in terms of measurement theory (McGrane and Maul Gevirtz, 2019).

From the earlier studies in this field, we know that the semantic structure usually emerges quite quickly with even a few respondents when it is as salient as in the present instrument (Arnulf et al., 2014a, 2018b). Sample sizes do not seem to be very crucial above a certain level. In the present case, the semantics predicted about equally well in the huge sample of Norwegians in Norwegian as in the much smaller samples such as Germans in German and English Natives. As expected, the Chinese samples seem to require a few more respondents for the matrix to approach the semantically given values. If some of our samples are below the optimal threshold for semantic predictability, increasing sample sizes would most likely increase the fit between semantic and respondent matrices.

Previous research has also indicated that groups of respondents display variance components from many sources, including personality and management level (Arnulf et al., 2018b). This is in accordance with what is expected from other studies on respondent characteristics in cross-cultural research (Harzing, 2006; He et al., 2014). This line of research asserts that differences between culturally divergent groups cannot be attributed to culture unless their respondent characteristics are controlled and accounted for. Our perspective is the opposite – we are simply aiming to show how much semantic patterns will unite proposedly different groups. Since our focus is on the extent of semantic influences, and since teasing apart variance components from the semantic structures requires more extensive laboratory work, this study has refrained from decomposing the origins of semantic structures further.

Taken together, our findings raise questions about the value of semantically driven surveys as a tool in cross-cultural leadership research methods. We believe that our data warrant the following three conclusions:

### Semantically Determined Surveys May Be Insensitive to Cultural Differences

The replication of data structures from semantically determined surveys may not tell us much about cultural differences, except for the fact that propositional structures in the survey have been correctly translated. This is a failure to distinguish between logical and empirical research questions (Russell, 1918/2007, 1922; Semin, 1989; Lovasz and Slaney, 2013; Smedslund, 2015; Arnulf et al., 2018b). The answers to logical research questions are given *a priori*, which is the reason why the response statistics are predictable by using computer algorithms that know nothing about respondents or cultures. This kind of research risks asserting that people and organizations are the same everywhere, disregarding the participants’ experiences that leadership phenomena are actually quite different across contexts (Henrich et al., 2010; Mendenhall, 2013). It is also likely to inflate statistics in ways that have frequently been demonstrated as effects of common method variance (Podsakoff et al., 2012; Schaller et al., 2015).

### Equivocality of Non-replication

Conversely, the main reason for observed differences in cases like the one we study here may simply be linguistic problems, either in the translation or in the respondents’ decoding of the item texts (Behr et al., 2016). The differences between samples in this study show that while the big bulk of relationships are semantically driven, there may be detectable differences that can masquerade as cultural differences because they are linked to different linguistic and ethnical groups. However, our findings also show that these differences may easily be explained by lack of language skills, local interpretations or faulty interpretations of the survey instrument. Even small differences in interpretations seem to influence the response statistics.

### Cross-Cultural OB Research Needs Better Philosophical Groundwork

The use of surveys in cross-cultural research on OB has for years avoided dealing with the difficult topic of what the “measurements” actually measure (Smedslund, 1988; Drasgow et al., 2015; Maul, 2017; Slaney, 2017). The original assumption of Likert (1932) was that the scales measure attitude strength, and that the ensuing statistical patterns were indicative of behavioral dispositions or inclinations. This assumption was originally doubted by his contemporaries in psychometrics, but Likert’s views prevailed as increasingly sophisticated statistical tools offered hopes of mathematical refinement (van Schuur and Kiers, 1994; Andrich, 1996). In recent years, though, the assumptions underlying measurements have come under renewed scrutiny. Some of the core psychometric criteria for construct validation are not capable of falsifying erroneous hypotheses, and the “measurements” may be measuring quite different entities from what they purport (Slaney and Racine, 2013; Mari et al., 2017; Maul, 2017; Arnulf et al., 2018b; Kjell et al., 2019).

The lack of awareness about these problems is all the more unfortunate in cross-cultural leadership, due to the risk of ethnocentrism inherent in the core problems of this field (Ng et al., 2009; Zhang et al., 2014; Ma and Tsui, 2015; Nagai et al., 2015). There is growing documentation about the fact that scholars as well as research subjects from “WEIRD” (White, Educated, Industrialized, Rich, and Democratic) countries are overwhelmingly represented with subsequent risks of theoretical and empirical biases (Henrich et al., 2010; Hibbing et al., 2014). Cross-cultural leadership is of great practical relevance in business and politics, and the costs of failures in this field are probably large (Gutierrez et al., 2012; Porter and Rivkin, 2012; Osland et al., 2013; Arvey et al., 2015). Anthropologists have for decades warned against the use of “thin data” in research on cross-cultural topics (Geertz, 1973).

When constructs like leadership are found to be semantically predictable to the extent found in this case, the most likely theoretical explanation is that it is precisely socially constructed (Berger and Luckman, 1966; Grint, 2005; Fairhurst and Grant, 2010). In this case, items may not so much be empirical “measures” as they are defining characteristics of the social construction (Smedslund, 1988; Elster, 2011, 2018; Lovasz and Slaney, 2013; Maul, 2017). The inter-item correlation matrix will then most likely reflect these mutual patterns in most languages whether the social construct is adopted in that culture or not.

The specific conclusion from this study is that cross-cultural studies in leadership need a more sophisticated view on the relationship between language and action in theory as well as practice. Studies that pick up semantic patterns are more likely to be language research than research on actions, a difference dealt with at length in action theory and control theory (Frese and Zapf, 1994; Weseman, 2007; Prinz et al., 2009; Parks-Stamm et al., 2010; Schaller et al., 2015; Gantman et al., 2017). When response patterns from semantically driven surveys are replicable across contexts, it may only mean that the same sentences can be said, with approximately the same understanding, across these contexts. This is unsurprising in itself – it equals the mere methodological requirement to have surveys translated and re-translated to ensure their identical meaning across languages (Herdman et al., 1997). In today’s global economies, most sentences that describe working environments may be translated from one language to another.

That is not the same as saying that the same things matter, that acts are carried out the same way, and with the same effects on people in the surroundings. The epistemological error that seems to be frequently committed in organizational behavior is to confuse behaviors with their intentions and effects on an abstract level. This has been theoretically proven by van Knippenberg and Sitkin (2013) in the case of transformational leadership, where definitions and operationalizations conflate independent and dependent variables.

Recent developments in indigenous Chinese research on leadership shows the likelihood that there exist distinct types of leadership behaviors that also have distinct effects on Chinese employees. This differs from the effects on, e.g., Western employees in the same companies (Chen and Kao, 2009; Cheng et al., 2014; Chen et al., 2015; Qin et al., 2015; Zhang et al., 2015). We obviously need more efforts to address the perceived differences that practitioners and scholars alike experience in the field, and generate instruments that capture these differences instead of neglecting them. That requires a less ethnocentric and more advanced philosophical foundation for understanding the role of language in research and cross-cultural leadership.

## Limitations and Suggestions for Future Research

The present study is a cross-sectional analysis of the responses to one single type of leadership survey. We believe that this is warranted, as we do not look at the temporal effects of the responses, but simply at the degree to which they are semantically determined. This means that the independent variable – the output from semantic algorithm – is not conflated with the dependent variables, i.e., the human responses. Also, we believe that the MLQ is an important exemplary type of leadership survey as it has been analyzed for its semantic structure in earlier publications and is a common instrument in cross-cultural leadership research.

The present study uses a series of mixed samples of various sizes and from various industries, locations and cultures. One clear limitation of our design is that the sub-samples are of unequal size and they are also not matched in terms of demographics and educational characteristics. We have no stringent control over the “cultural” diversity in the samples except for the locations and the languages of the respondents. We do think that our design goes a long way to randomize factors like industries, professions and other non-intended sample characteristics. Still, there may be better methods to control and document the cultural conditions that are central in determining differences in leadership.

One particular limitation of the present study is that we have only used English language items to inform the algorithms. As expected, the ability of the algorithms to predict response patterns were better for English and linguistically related groups than for groups with cultures and languages more distant to English. Our design can for good reason be suspected of adopting a culturally skewed perspective in the algorithms themselves. As explained, we believe this is warranted as a first step here due to the WEIRD heritage of the leadership constructs and measurement instruments themselves. The semantic perspective raises a question about how indigenous, non-WEIRD leadership issues should be conceptualized both as theoretical constructs and as measurements. Further developments in this field are necessary to create a viable research agenda here.

Finally, this study did not look specifically at cultural differences in score levels between cultures. We do think that more valid information about cross-cultural leadership research can be found in that direction. This study has concentrated on studying the relationships between item pairs and subscales, as these are frequently used as important inputs for further statistical modeling.

For future research, we highly recommend more controlled studies where the semantic influences on survey statistics are more clearly identified as sources of variation. We know that attempts at using multi-trait multi-method approaches are under way (Martinsen et al., 2017). It is imperative that the semantic components are identified and properly understood, for example as sources of common method variance (Bagozzi, 2011) or as a general response style (He et al., 2014). To truly understand the unique impact of semantic relationships in cross-cultural research, we nee more knowledge about high-quality instruments with balanced items, so that the effect of item types on the semantic structure would be easier to discern.

## Data Availability Statement

The datasets generated for this study are available on request to the corresponding author.

## Ethics Statement

The studies involving human participants were reviewed and approved by Norsk Samfunnsvitenskapelig Datatjeneste. The patients/participants provided their written informed consent to participate in this study.

## Author Contributions

JA designed the study, collected the survey data materials, and co-wrote the manuscript. KL provided the semantic algorithms, helped develop the theory, and co-wrote the manuscript.

## Conflict of Interest

The authors declare that the research was conducted in the absence of any commercial or financial relationships that could be construed as a potential conflict of interest.

## Footnotes

1 http://www.mindgarden.com/
